# Novel defatting strategies reduce lipid accumulation in primary human culture models of liver steatosis

**DOI:** 10.1242/dmm.042663

**Published:** 2020-04-29

**Authors:** Lynda Aoudjehane, Jérémie Gautheron, Wilfried Le Goff, Claire Goumard, Julia Gilaizeau, Chan Sonavine Nget, Eric Savier, Muhammad Atif, Philippe Lesnik, Romain Morichon, Yves Chrétien, Yvon Calmus, Olivier Scatton, Chantal Housset, Filomena Conti

**Affiliations:** 1 Institute of Cardiometabolism and Nutrition (ICAN), Sorbonne Université, INSERM, Paris 75012, France; 2 Centre de Recherche Saint-Antoine (CRSA), Sorbonne Université, INSERM, Paris 75013, France; 3 Department of Hepatobiliary and Liver Transplantation Surgery, Pitié-Salpêtrière Hospital, Assistance Publique-Hôpitaux de Paris, Paris 75013, France; 4 Centre d'immunologie et maladies infectieuses, Sorbonne Université, INSERM, U1135, Paris 75013, France; 5 Production et Analyse des données en Sciences de la vie et en Santé (PASS), Sorbonne Université, INSERM, UMS 37, Paris 75013, France; 6 Department of Medical Liver Transplantation, Pitié-Salpêtrière Hospital, Assistance Publique-Hôpitaux de Paris, Paris 75013, France; 7 Department of Hepatology, Reference Center for Inflammatory Biliary Diseases and Autoimmune Hepatitis, Saint-Antoine Hospital, Assistance Publique-Hôpitaux de Paris, Paris 75012, France

**Keywords:** Defatting, Human hepatocytes, Human precision-cut liver slices, Liver transplantation, Steatosis, Triglycerides

## Abstract

Normothermic perfusion provides a means to rescue steatotic liver grafts, including by pharmacological defatting. In this study, we tested the potential of new drug combinations to trigger defatting in three human culture models, primary hepatocytes with induced steatosis, primary hepatocytes isolated from steatotic liver, and precision-cut liver slices (PCLS) of steatotic liver. Forskolin, L-carnitine and a PPARα agonist were all combined with rapamycin, an immunosuppressant that induces autophagy, in a D-FAT cocktail. D-FAT was tested alone or in combination with necrosulfonamide, an inhibitor of mixed lineage kinase domain like pseudokinase involved in necroptosis. Within 24 h, in all three models, D-FAT induced a decrease in triglyceride content by 30%, attributable to an upregulation of genes involved in free fatty acid β-oxidation and autophagy, and a downregulation of those involved in lipogenesis. Defatting was accompanied by a decrease in endoplasmic reticulum stress and in the production of reactive oxygen species. The addition of necrosulfonamide increased the efficacy of defatting by 8%-12% in PCLS, with a trend towards increased autophagy. In conclusion, culture models, notably PCLS, are insightful to design strategies for liver graft rescue. Defatting can be rapidly achieved by combinations of drugs targeting mitochondrial oxidative metabolism, macro-autophagy and lipogenesis.

## Abstract (precis)

**Summary:** This study describes a defatting cocktail that has been proven to function in three relevant steatotic human culture models without cytotoxicity, and which could be employed in the reduction of steatosis in donor livers during liver transplantation.

## INTRODUCTION

The ongoing donor-organ supply mismatch is a major barrier to reducing the high mortality rate of patients on the waiting list for a liver transplant. Excessive liver steatosis is a key reason for discarding the donor graft. This is because the presence of steatosis increases the risk of immediate liver graft dysfunction, and of a suboptimal outcome post-transplantation (Henry et al., 2012).

Several strategies, primarily based on machine perfusion, have been recently developed to rescue marginal liver grafts (Kollmann and Selzner, 2017; Kron et al., 2017). This is particularly interesting from the perspective of steatotic liver grafts, as pharmacological agents can be added to the perfusion circuit during normothermic perfusion to stimulate a defatting process. Indeed, this strategy has been previously demonstrated in preclinical models (Nagrath et al., 2009). This latter group and others have used different pharmacological modulators of triglyceride (TG) metabolism combined with other drugs to reduce the lipid content in cultured rat fatty hepatocytes, in isolated perfused rat fatty livers (Nagrath et al., 2009; Nativ et al., 2013) and, more recently, in fat-loaded primary human hepatocytes (PHH) (Boteon et al., 2018).

In the present study, we developed a novel defatting cocktail by combining drugs similar to those included in previous cocktails (Nagrath et al., 2009), i.e. forskolin (20 µmol/l), L-carnitine (1 mmol/l), PPARα agonist (GW7647, 1 µmol/l), as well as others that target other pathways involved in fat storage, i.e. rapamycin (200 nmol/l) and necrosulfonamide (NSA, 10 µM). We then investigated the beneficial effects of the cocktail and the mechanisms of action using a human hepatocyte primary culture in which steatosis was induced, PHH which were isolated from human fatty liver samples, and an *ex vivo* 3D model: human precision-cut liver slices (hPCLS) obtained from fatty liver samples. In this study, we have generated and used, for the first time, viable and functional steatotic hPCLS, a relevant *ex vivo* preclinical model that retains the complex and multi-cellular histoarchitecture of the human hepatic environment.

We propose a well-designed defatting cocktail composed of agents demonstrated to significantly reduce TG content in fatty livers. Forskolin stimulates lipolysis of TG, leading to the generation of free fatty acids (FFA): FFA serve as substrates for β-oxidation in mitochondria. L-carnitine is a substrate of carnitine palmitoyltransferase 1A (CPT1A), a gatekeeper enzyme for the entry of long-chain fatty acids into mitochondria and their oxidation. The role of PPARα agonists is to induce the expression of target genes encoding enzymes or proteins, including CPT1A, microsomal triglyceride transfer protein (MTTP), a key enzyme in very-low-density-lipoprotein (VLDL) production, which catalyses the transfer of TG to apolipoprotein B (APOB100) and apolipoprotein A1 (APOA1), which are involved in cholesterol export. In addition, we also used the immunosuppressant rapamycin, which can decrease steatosis by inhibiting mammalian target of rapamycin (mTOR), which promotes lipogenesis, the induction of TG secretion and macro-autophagy (Lin et al., 2013; Waskowicz et al., 2019; Zhou and Ye, 2018). Finally, we tested NSA in our cocktail, which is a specific inhibitor of the mixed lineage kinase domain like pseudokinase (MLKL), an effector of the necroptosis pathway, which recently emerged as a regulator of insulin sensitivity and TG storage in the liver (Xu et al., 2019).

## RESULTS

### Model of FFA-induced steatosis in PHH

**Fig. 1.**
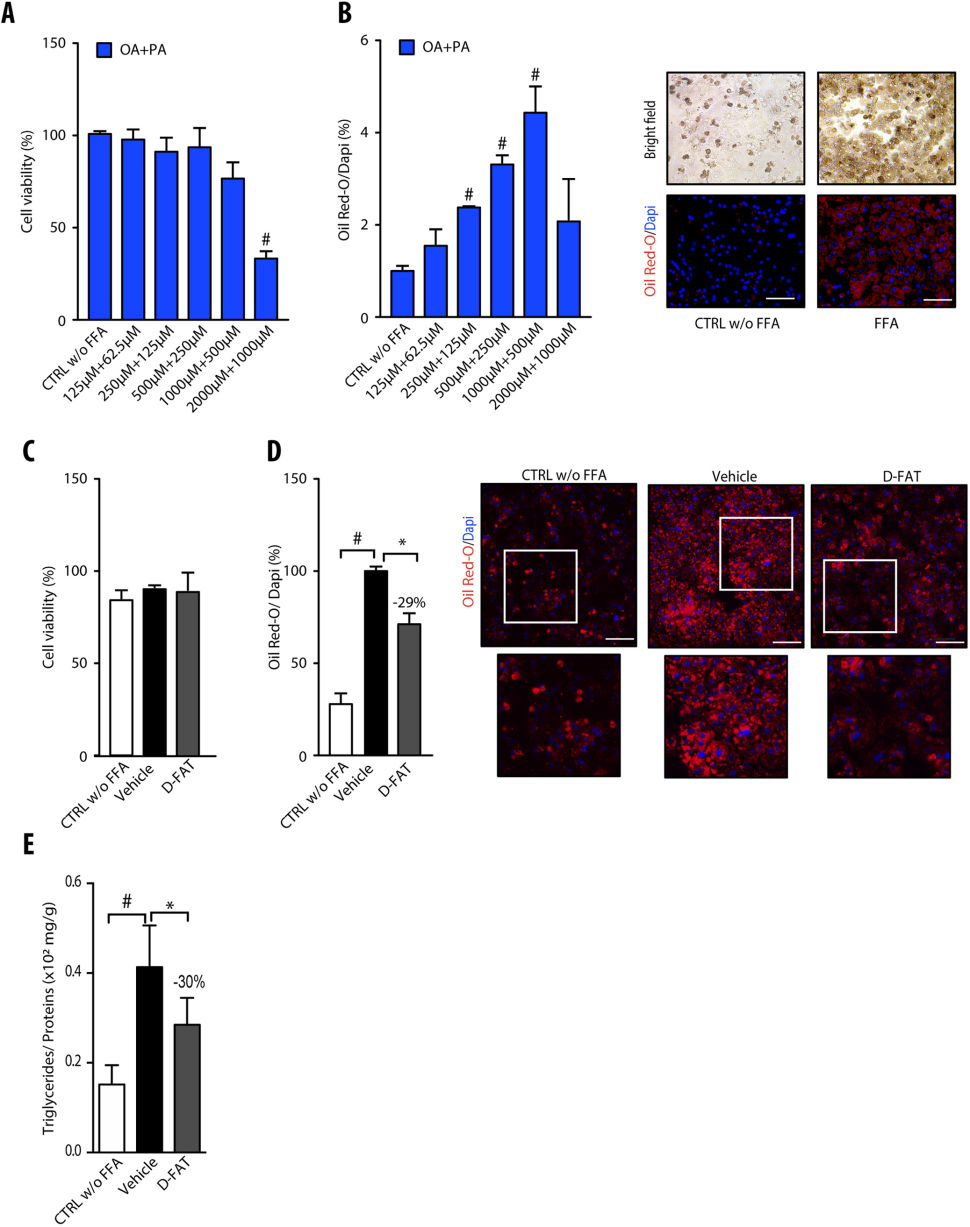
**Steatosis induction and defatting response to D-FAT in PHH**. (A,B) Normal human hepatocytes in primary culture were incubated with or without different concentrations of the free fatty acid (FFA) mixture oleic acid (OA) and palmitic acid (PA) (2:1) for 48 h, and examined for (A) cell viability, assessed by MTT assay, and (B) lipid droplet content, assessed by Oil Red O staining. In B, left panel shows quantification of Oil Red O staining, normalized to the number of DAPI-stained nuclei; right panel shows representative images of controls (CTRL) and FFA-loaded PHH (OA:PA, 500:250 μmol/l). (C-E) Normal human hepatocytes in primary culture were incubated with or without FFA mixture (OA:PA, 500:250 μmol/l) for 48 h, and thereafter FFA-loaded PHHs were treated with the D-FAT cocktail or the vehicle for 24 h, and then subjected to (C) cell viability, assessed via the MTT assay; or assessed for (D) lipid droplet content, by Oil Red O staining; or (E) intracellular triglyceride (TG) content normalized for cell protein. In D, left panel shows quantification of Oil Red O staining; right panel shows representative images of controls and FFA-loaded PHH at baseline, and after vehicle or DFAT treatment (lower panels show magnification of boxed areas in upper panels). Means±s.e.m. of six cell preparations are shown relative to controls in A and B and to vehicle in D and E. In all panels, ^#^*P*<0.05 versus control, *P<0.05 versus vehicle (one-way ANOVA). Scale bars: 50 μm.

Normal hepatocytes in primary culture were incubated with different concentrations of the FFA mixture oleic acid (OA):palmitic acid (PA) in the molar ratio 2:1, for 48 h, to induce steatosis. Up to concentrations of 1000:500 µmol/l, incubation with OA:PA did not significantly affect cell viability, as shown by MTT assay (Fig. 1A). The quantification of Oil Red O-stained areas showed that, up to this concentration ratio, treatment with FFAs induced a dose-dependent accumulation of fat in hepatocytes (Fig. 1B). Hence, the OA:PA concentration ratio of 500:250 µmol/l that increased the lipid droplet content by approximately threefold within 48 h, without affecting cell viability, was selected to induce steatosis in subsequent experiments.

### Effects of D-FAT cocktail in PHH with FFA-induced steatosis

The D-FAT cocktail contains drugs that are all approved, and the targets of which modulate metabolic pathways in a complementary manner. We also added rapamycin to inhibit mTOR and, thereby, macro-autophagy. Of particular interest in this respect, PHH with FFA-induced steatosis displayed a decrease in the expression of the genes encoding microtubule associated proteins 1A/1B light chain 3 beta (LC3; also known as MAP1LC3B) and sirtuin-1 (SIRT1), two markers of autophagy activation (Tong et al., 2016) (Fig. S1A,B). The treatment with rapamycin could activate autophagy induction, increasing the expression of LC3 and SIRT1 (Fig. S1C-E).

Compounds of the D-FAT cocktail were first tested individually for cytotoxicity and lipid decrease. No overall effect on PHH viability was found (Fig. S2A). The content of intracellular lipid droplets was determined by Oil Red O staining and was not significantly decreased following individual treatment with each compound (Fig. S2B). Therefore, intracellular TG content was not significantly decreased with compounds of D-FAT tested individually, except for rapamycin, which reduced intracellular TG by 18% (*P*<0.05) compared with vehicle control (Fig. S2C).

To test the effect of the D-FAT cocktail on steatosis, PHHs with FFA-induced steatosis were treated with the cocktail for 24 h. The intracellular lipid content was assessed by Oil Red O staining and TG assay. The treatment with D-FAT was non-cytotoxic (Fig. 1C) and caused a significant decrease in intracellular lipid droplets (Fig. 1D). Oil Red O-stained area was reduced by 29% compared with vehicle-treated steatotic hepatocytes (Fig. 1D). Likewise, intracellular TG content was decreased by 30% (*P*<0.05) in D-FAT-treated hepatocytes compared with vehicle-treated steatotic cells (Fig. 1E).

**Fig. 2.**
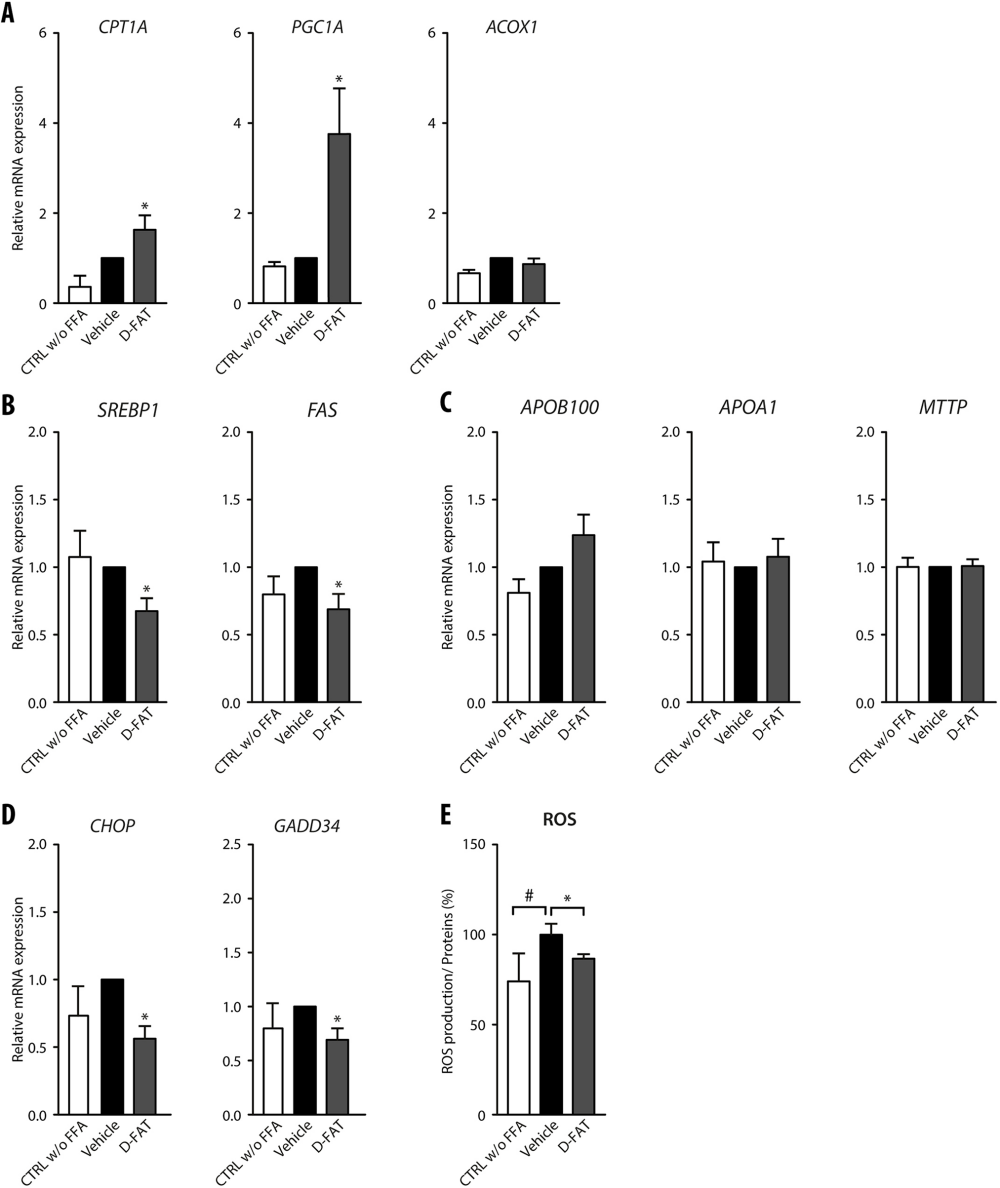
**Pathways altered along with defatting in response to D-FAT in PHH.** (A-E) Normal human hepatocytes in primary culture were incubated with or without FFA mixture (OA:PA, 500:250 µmol/l) for 48 h, and thereafter FFA-loaded PHHs were treated with the D-FAT cocktail or the vehicle for 24 h. PHHs in all conditions were subjected to RT-qPCR analyses of genes involved in fatty acid β-oxidation (A) (*CPT1A*, *PGC1A*, *ACOX1*); lipogenesis (B) (*SREBP1*, *FAS*); lipid export (C) (*ApoB100*, *ApoA1*, *MTTP*); and ER stress (D) (*CHOP*, *GADD34*). (E) Measurement of intracellular ROS by spectrophotometric quantification of H2DCFDA. Data are mean±s.e.m. of six cell preparations, shown relative to vehicle. ^#^*P*<0.05 versus control, *P<0.05 versus vehicle (one-way ANOVA).

We then sought to identify the underlying mechanisms to explain how defatting occurred in D-FAT-treated steatotic hepatocytes. From the perspective of genes involved in fatty acid β-oxidation, we found that *CPT1A* and peroxisome proliferator-activated receptor-γ coactivator 1 alpha (*PGC1A*; also known as *PPARGC1A*) genes were significantly upregulated in steatotic hepatocytes treated with the D-FAT cocktail (Fig. 2A). However, no difference was observed in *ACOX1* gene expression regardless of treatment condition (Fig. 2A). Ketone bodies that are produced as a result of fatty acid β-oxidation showed a trend towards increased secretion in the supernatant of D-FAT-treated steatotic hepatocytes, although the difference with vehicle-treated cells was not statistically significant (*P*=0.05) (Fig. S3A). Furthermore, the expression of genes involved in lipogenesis, such as sterol regulatory element binding protein-1 (*SREBP1*; *SREBF1*) and fatty acid synthase (*FAS*; *FASN*), was significantly decreased following D-FAT treatment in steatotic hepatocytes (Fig. 2B). We also assessed the export pathways and found a trend towards increased amounts of TG and apolipoprotein B100 (APOB100) secreted in the cell supernatant of D-FAT-treated steatotic hepatocytes (Fig. S3B,C). Also, the expression of APOB100, APOA1 and MTTP was not significantly different between D-FAT- and vehicle-treated steatotic hepatocytes (Fig. 2C).

Hepatocellular features associated with steatosis include endoplasmic reticulum (ER) stress, oxidative stress and a pro-inflammatory phenotype. We sought to determine whether some of these features could be attenuated, along with fat depletion, in hepatocytes treated with the D-FAT cocktail. ER stress causes an activation of the protein kinase R-like endoplasmic reticulum kinase (PERK) pathway and overexpression of its downstream targets, CCAAT-enhancer-binding protein homologous protein (CHOP; DDIT3) and growth arrest and DNA damage-inducible protein 34 (GADD34; PPP1R15A). Indeed, we found that CHOP and GADD34 underwent a significant downregulation in their expression in steatotic PHHs that were treated with the D-FAT cocktail (Fig. 2D). Moreover, we found that the production of reactive oxygen species (ROS) increased alongside the accumulation of fat in PHHs following their incubation with FFA, whereas the production of ROS returned to basal levels following treatment with the D-FAT cocktail and was significantly lower in D-FAT-treated steatotic PHHs compared with vehicle-treated cells (Fig. 2E).

### Efficacy of D-FAT cocktail in PHHs isolated from human fatty livers

**Fig. 3.**
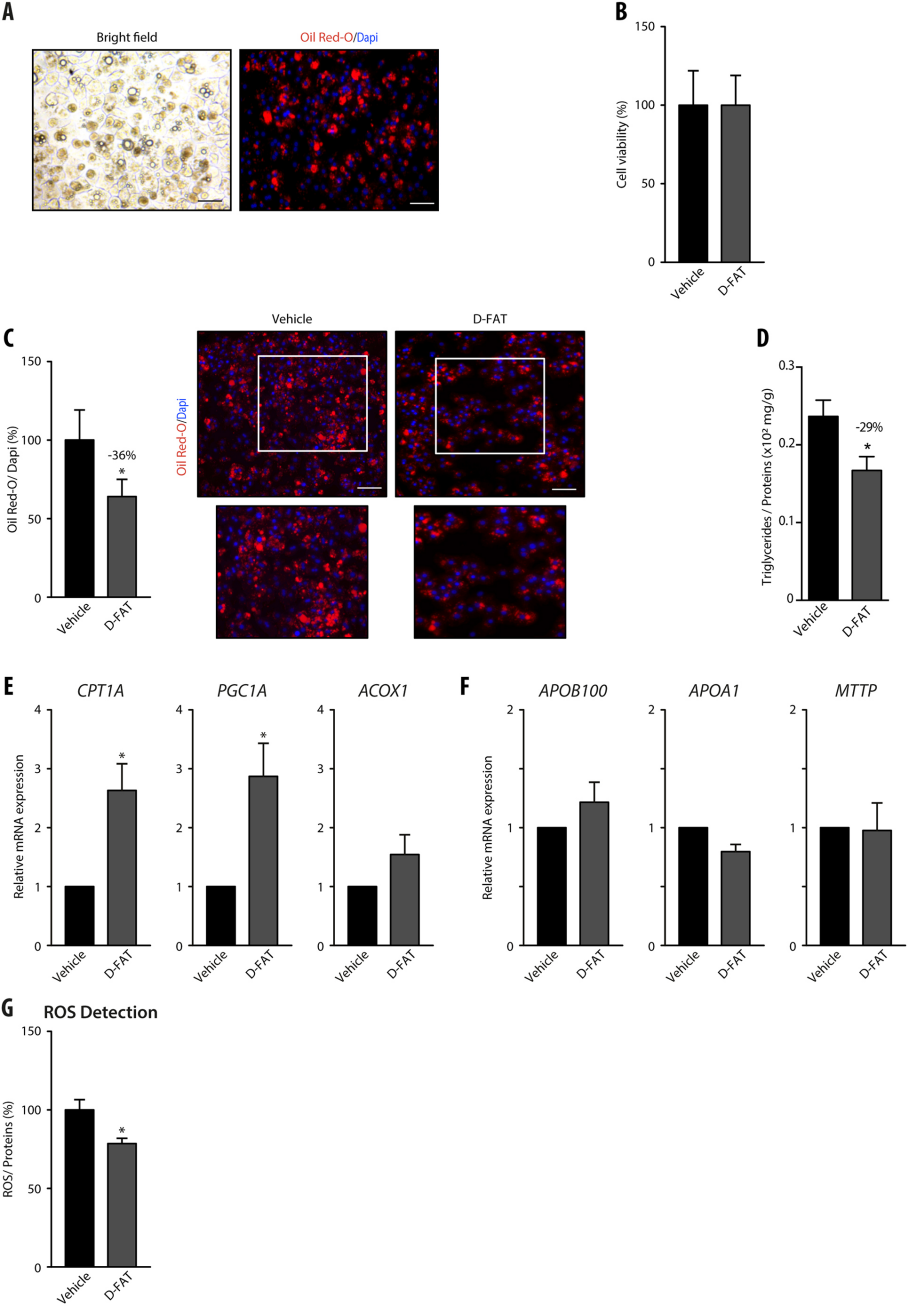
**Efficacy of the D-FAT cocktail on steatotic hepatocytes isolated from human fatty liver.** (A-G) Hepatocytes were isolated from steatotic human liver samples. After 24 h in primary culture their lipid droplet content was assessed by Oil Red O staining (A). Following treatment with the D-FAT cocktail or vehicle for 24 h, samples were examined for: (B) cell viability, assessed by MTT assay; (C) lipid droplet content, assessed by Oil Red O staining – right panels show representative images of vehicle- and D-FAT-treated steatotic hepatocytes (lower panels show magnification of boxed areas in upper panels), left panel shows quantification of Oil Red O staining, normalized to the number of DAPI-stained nuclei; (D) intracellular TG content normalized to cell protein; (E) RT-qPCR analyses of genes associated with fatty acid β-oxidation (*CPT1A*, *PGC1A*, *ACOX1*); (F) RT-qPCR analyses of genes associated with lipid export (*ApoB100*, *ApoA1* and *MTTP*). (G) Measurement of intracellular ROS. Data are mean±s.e.m. of six cell preparations, shown relative to vehicle. *P<0.05 versus vehicle (two-tailed Student's *t*-test). Scale bars: 50 µm.

To confirm the efficacy of pharmacological defatting on steatosis developed *in vivo*, we tested the D-FAT cocktail on steatotic hepatocytes that were isolated from human fatty livers. Hepatocytes isolated from fatty livers remained steatotic after 24 h in primary culture (Fig. 3A). Akin to FFA-induced steatotic PHHs, these cells treated with the D-FAT cocktail also displayed no cytotoxicity (Fig. 3B) and underwent a significant depletion in their lipid content (Fig. 3C,D). After incubation with the D-FAT cocktail for 24 h, Oil Red O-stained area and intracellular TG content were reduced by 36% and 29%, respectively, compared with vehicle-treated cells (Fig. 3C,D). In this model, as previously mirrored by FFA-induced steatotic PHHs, the cocktail also induced an upregulation of genes involved in fatty acid β-oxidation (Fig. 3E) but had no significant effect on the expression of genes involved in lipid export (Fig. 3F). Also, treatment with the D-FAT cocktail significantly reduced the production of ROS (Fig. 3G).

### Efficacy of D-FAT and its combination with NSA, in steatotic PHH and PCLS

In addition to its role as an effector of the necroptosis pathway, MLKL has recently emerged as a regulator of insulin sensitivity and hepatic TG storage (Xu et al., 2019). We hypothesized that we could supplement our defatting strategy by also targeting MLKL. In support of this, we found that the expression of MLKL was upregulated in PHH cultured in steatosis-inducing conditions, but its expression returned to basal levels when these cells were treated with NSA, an MLKL inhibitor (Fig. S4A). In parallel, the lipid content and intracellular TG were investigated in FFA-induced steatotic PHHs treated with NSA for 24 h. The treatment with NSA was not cytotoxic (Fig. S4B), but it caused a significant decrease in intracellular lipid droplets (17%; *P*<0.05; Fig. S4C) and intracellular TG (19%; *P*<0.05; Fig. S4D) compared with vehicle-treated steatotic hepatocytes.

**Fig. 4.**
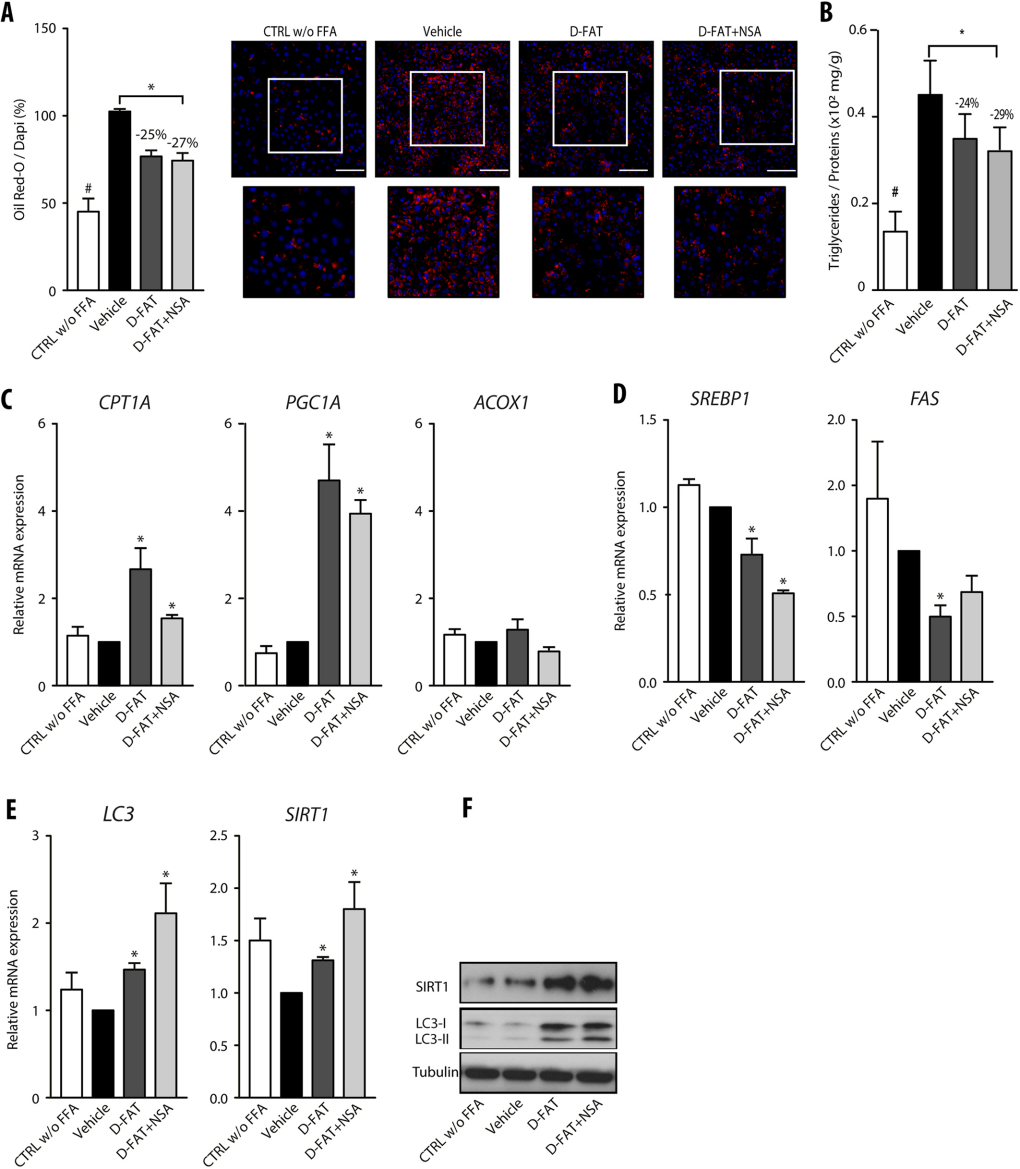
**Combined effect of NSA with the D-FAT cocktail on defatting in fat-loaded PHH.** (A-F) Normal human hepatocytes in primary culture were incubated with or without FFA mixture (OA:PA, 500:250 µmol/l) for 48 h, and thereafter FFA-loaded PHH were treated with D-FAT, D-FAT+NSA or vehicle for 24 h. (A) Quantification of lipid droplet content, assessed by Oil Red O staining. Left panel shows quantification of Oil Red O staining, normalized for the number of DAPI-stained nuclei; right panels show representative images (lower panels show magnification of boxed areas in upper panels). (B) Quantification of intracellular TG content normalized for cell protein. (C,D) RT-qPCR analyses of genes involved in fatty acid β-oxidation (C) (*CPT1A*, *PGC1A*, *ACOX1*) and lipogenesis (D) (*SREBP1*, *FAS*). (E,F) Genes involved in autophagy (*LC3*, *SIRT1*) were examined by RT-qPCR analyses (E) and western blotting (F). Data are mean±s.e.m. of six cell preparations, shown relative to the vehicle. *P<0.05 versus vehicle (one-way ANOVA). Scale bars: 50 µm.

Furthermore, the addition of NSA to the D-FAT cocktail induced a trend towards enhanced fat depletion in FFA-induced steatotic PHHs, as shown by Oil Red O staining and intracellular TG content, although the differences with D-FAT alone were not significant (Fig. 4A,B). Among the pathways of defatting that we assessed using reverse transcription-quantitative polymerase chain reaction (RT-qPCR) analyses (Fig. 4C-E), we also identified a trend towards a higher upregulation of LC3 and SIRT1 in response to the combination of D-FAT with NSA compared with D-FAT alone (Fig. 4E,F) and these results were confirmed by western blotting (Fig. 4F).

**Fig. 5.**
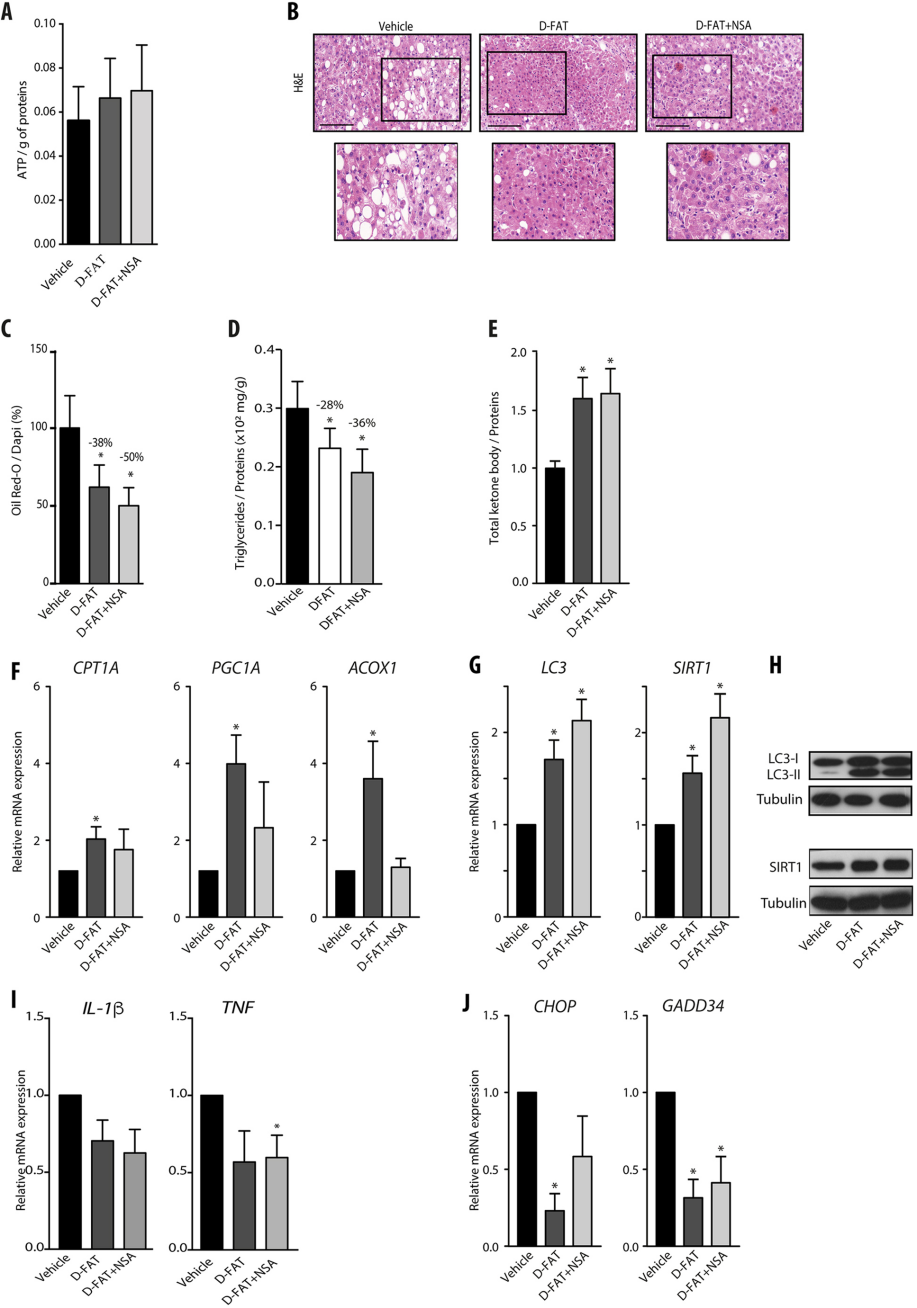
**Efficacy of defatting cocktails in human steatotic PCLS.** (A-J) PCLS were prepared from steatotic human liver samples, and after 1 h in primary culture they were treated with D-FAT, D-FAT with NSA, or vehicle for 24 h, and examined for: (A) cell viability assessed by ATP content; (B) histology of H&E-stained tissue sections – representative images are shown (lower panels show magnification of boxed areas in upper panels); (C) lipid droplet content, assessed by Oil Red O staining; (D) intracellular TG content; (E) ketone bodies secreted in the cell supernatants. (F) RT-QPCR analyses of genes involved in fatty acid β-oxidation (*CPT1A*, *PGC1A*, *ACOX1*). (G,H) RT-QPCR analyses of genes (G) and western blotting of proteins (H) involved in autophagy induction (*LC3*, *SIRT1*). (I,J) RT-QPCR analyses of pro-inflammatory interleukin genes (*IL1B*, *TNF*) (I) and of genes involved in ER stress (J) (*CHOP*, *GADD34*). Data are mean±s.e.m. of seven preparations, shown relative to vehicle. *P<0.05 versus vehicle (one-way ANOVA). Scale bars: 100 µm.

To address the feasibility of defatting by D-FAT and the added value of NSA on intact liver tissue, we used PCLS, a model in which the 3D structure of liver tissue and the interactions of hepatocytes with other liver cell types are maintained as in the whole organ. This model was feasible as cell viability in PCLS was stable up until 48 h in culture, as ascertained by histological integrity and ATP intracellular content (Fig. S5A,B). After 1 h of culture, PCLS sourced from human fatty livers were incubated with D-FAT cocktail with or without NSA, or with the vehicle, for 24 h. We found that the D-FAT cocktail (with or without NSA) did not alter PCLS viability and, on the contrary, caused a trend towards increased ATP content (Fig. 5A). Combined or not with NSA, the D-FAT cocktail provoked a marked depletion in lipid droplets (Fig. 5B). The live imaging videos of Oil Red O-stained PCLS of fatty livers also showed that, when compared with vehicle-treated PCLS (Movie 1), the abundance of lipid inclusions was substantially decreased in D-FAT-treated PCLS (Movie 2), and to an even greater extent in those treated with D-FAT combined with NSA (Movie 3). The Oil Red O-stained area was significantly reduced in PCLS treated with D-FAT with or without NSA, by 38% and 50%, respectively, compared with the vehicle (*P*<0.05) (Fig. 5C). The results were confirmed by the significant decrease in TG content, which was reduced by 28% and 36% when the vehicle was compared to D-FAT with or without NSA, respectively (Fig. 5D). In this model, both cocktails, i.e. D-FAT combined or not with NSA, caused a significant increase in the ketone bodies released in the culture supernatant compared with the vehicle control (Fig. 5E). Put together, these data indicated that, although both conditions stimulated fatty acid β-oxidation, only D-FAT without NSA induced a significant increase in the expression of genes mediating fatty acid β-oxidation (Fig. 5F). Moreover, the expression of LC3 and SIRT1 implicated in autophagy was also increased by both cocktails, with a trend towards a higher increase induced by the combination of D-FAT with NSA, as in PHHs (Fig. 5G,H). The steatosis was also accompanied by an increase in the expression of pro-inflammatory interleukins (IL), IL-1β and TNF in PCLS. The mRNA levels of cytokines decreased, but not significantly compared with vehicle, except for TNF, which was significantly reduced by D-FAT in combination with NSA on treated PCLS (Fig. 5I). Finally, we also found that CHOP expression was significantly downregulated by D-FAT, and the expression of GADD34 in PCLS of human fatty liver was significantly decreased by both cocktails, indicating that both conditions reduced ER stress too (Fig. 5J).

## DISCUSSION

In order to increase the donor pool for liver transplantation, marginal livers, including steatotic livers, are increasingly being used. Although steatotic livers already make up a major proportion of discarded livers, we predict that this proportion will increase in the future owing to the global obesity epidemic. Although graft steatosis is reversible post-transplantation, it nevertheless predisposes to graft dysfunction and drastically reduces the survival of the graft. Hence, a proposed solution to mitigate these complications has been to treat steatotic grafts before transplantation with defatting solutions during normothermic perfusion. The feasibility of this approach has been recently demonstrated in humans whereby pharmacological modulation of lipid metabolism during normothermic perfusion decreased the lipid content of liver grafts (Boteon et al., 2019). In our study, we developed a defatting solution based on previously tested drugs that we supplemented with agents targeting additional pathways involved in the development of non-alcoholic fatty liver disease (NAFLD) such as autophagy and necroptosis.

The first finding in our study was the ability of the D-FAT cocktail, which contains only on-market drugs, to significantly defat steatotic human hepatocytes mainly via activating the β-oxidation pathway. This cocktail combines drugs that have already been shown to defat steatotic hepatocytes within 48 h by activating lipid metabolic pathways, as well as an additional drug, rapamycin, which we anticipated would augment the defatting process. The addition of rapamycin was justified based on previous data demonstrating its role in amplifying fatty acid oxidation (Brown et al., 2007), TG export (Mathe et al., 1992) and autophagy induction (Martínez-Cisuelo et al., 2016; Nakadera et al., 2016). In addition, target of rapamycin complex 1 (mTORC1) positively regulates sterol regulatory element binding proteins, stimulates lipogenesis (Chen et al., 2018) and promotes fat storage (Bakan and Laplante, 2012; Chakrabarti et al., 2010).

The models that were used in this study are relevant preclinical models of human liver transplantation. PHH are more appropriate to study lipid synthesis and secretion compared with hepatoma cell lines or rodent primary hepatocyte models (Ling et al., 2013). The first model of steatotic human hepatocytes was based on incubating PHHs with an FFA mixture for 48 h. OA and PA are the most abundant FFAs in liver TG in both healthy individuals and patients with NAFLD (Gori et al., 2016; Baylin et al., 2002). Moreover, as the OA:PA mixture we used caused minor cytotoxicity, the remaining steatotic hepatocytes after exposure to this mixture could be considered as a cell model of benign liver steatosis (Gómez-Lechón et al., 2007). We also used two other models that even more accurately reflect *in vivo* liver steatosis: PHH isolated from steatotic livers and human steatotic PCLS. In the present study, we show, for the first time to our knowledge, that human steatotic PCLS remain functional and viable, with high levels of ATP, for at least 48 h in culture. This latter model, which retains the complex multi-cellular histoarchitecture of the hepatic environment and many biological functions of the liver (Palma et al., 2019), is likely the most relevant to assess the efficacy and the mechanisms of defatting strategies.

In human steatotic PCLS as in the other models, the cocktails developed by us consistently caused a significant reduction in lipid droplets and intracellular TG without affecting cell viability after 24 h of incubation. This time period would be compatible with clinical use of our cocktail in a perfusion fluid aiming to rescue steatotic liver grafts. From a mechanism perspective, our defatting process mainly involved activation of β-oxidation and the inhibition of lipogenesis as well as a minor involvement of increased TG export. We showed that D-FAT treatment caused no change in the secretion of ApoB100, an essential component of VLDL, but increased the secretion of ketone bodies, an index of β-oxidation. Consistent with these results, the expression of CPT1A and PGC1α, enzymes involved in fatty acid β-oxidation, was upregulated following D-FAT treatment, whereas the expression of MTTP and ApoB100 was unchanged, and that of SREBP1 and FAS was reduced. Lipid accumulation in hepatocytes is associated with ER stress, oxidative stress and inflammation (Machado and Cortez-Pinto, 2014), explaining the increased sensitivity of steatotic liver grafts to ischemia-reperfusion injury. Indeed, our data show that ROS, which were elevated in steatotic hepatocytes and human steatotic PCLS, were reduced by D-FAT treatment. D-FAT treatment was also associated with an inhibition of ER stress. Therefore, we conclude that the D-FAT cocktail exerts additional beneficial effects, which, during liver graft perfusion, may also attenuate ischemia-reperfusion injury.

In order to improve the effectiveness of the defatting process, we targeted necroptosis, a newly described pathway in the development of NAFLD. Recent studies have shown that MLKL-dependent necroptosis is activated in different types of liver injury, including NAFLD (Gautheron et al., 2016). In the present study, we showed that MLKL expression was increased in fat-loaded human hepatocytes compared with controls and returned to basal levels when exposed to our cocktail containing the MLKL inhibitor, NSA. Moreover, the addition of NSA to the D-FAT cocktail increased the efficacy of defatting, as demonstrated by 3D-imaging of the human steatotic PCLS. Although the exact mechanisms of this effect remain to be fully elucidated, our data suggest it is at least partly mediated by increased autophagy.

In conclusion, by targeting different pathways involved in TG homeostasis, our D-FAT cocktail reduces the amount of intracellular fat droplets and TG content by 25% to 30% within 24 h in steatotic human hepatocytes and PCLS. The underlying mechanisms mainly involve increased β-oxidation, decreased lipogenesis and also likely activation of macro-autophagy. This latter effect was boosted by the addition of an MLKL inhibitor, which further increased the efficacy of defatting. The D-FAT cocktail reduces ER stress and oxidative stress that occur in liver steatosis, which may ameliorate liver injury during the preservation process. Although normothermic perfusion of liver grafts has not yet exceeded 24 h in clinical trials, it is likely that graft perfusion for longer durations will be possible in the future. This would allow the D-FAT and related cocktails to induce even more profound defatting and improve the quality of the transplanted graft.

## MATERIALS AND METHODS

### Ethics statement

Ethical approval for the study was granted by the Persons Protection Committee (CPP Ile de France III) and by the French Ministry of Health (Ref COL 2929 and COL 2930). Liver tissue was obtained from patients who underwent surgery at Assistance Publique-Hôpitaux de Paris Hospitals, Paris, France. All patients gave informed consent, all clinical investigation was conducted according to the principles expressed in the Declaration of Helsinki and all samples were anonymized. Cell isolation and PCLS preparation were performed on the Human HepCell platform (ICAN, Paris, France; http://www.ican-institute.org/category/plateformes) in compliance with ethical guidelines.

### Isolation and culture of PHHs

Histologically normal and steatotic liver tissue samples (*n*=28) were obtained from subjects including 57.5% (*n*=16) females and 42.5% males (*n*=12), mean age 55.10±11.7 years old undergoing partial hepatectomy for the treatment of colorectal cancer metastases or primary liver cancer. In this study, 14 samples were from steatotic livers, with 20% to 60% steatotic hepatocytes. All samples were seronegative for the hepatitis C virus (HCV), the hepatitis B virus (HBV) and the human immunodeficiency virus (HIV). PHH were prepared as previously described (Aoudjehane et al., 2007; Aoudjehane et al., 2016). The liver fragment was initially perfused with a pre-warmed (37°C) calcium-free buffer supplemented with 5 mmol/l ethylene glycol tetraacetic acid (Sigma-Aldrich) followed by perfusion with a pre-warmed (37°C) buffer containing 6 mmol/l calcium (CaCl_2_) and collagenase 0.05% (5 mg/ml) (Sigma-Aldrich). The liver fragment was then gently shaken to disperse liver cells in Hepatocyte Wash Medium (Life Technologies). The resulting cellular suspension was filtered through a gauze-lined funnel. Cells were then centrifuged at low speed (50 ***g***). The supernatant, containing damaged or dead hepatocytes, non-parenchymal cells and debris was removed and pelleted hepatocytes were re-suspended in Hepatocyte Wash Medium. The count of viable cells was determined using Trypan Blue exclusion. Freshly isolated normal or steatotic hepatocytes were suspended in William's E medium (Life Technologies) containing 10% fetal calf serum (Eurobio), penicillin (200 U/ml)-streptomycin (200 µg/ml; Life Technologies), fungizone (2.5 µg/ml; Life Technologies) and insulin (0.1 U/ml; Sigma-Aldrich). The cells were then seeded in 12-, 24- and 96-well plates pre-coated with type I collagen at a density of 0.78×10^6^, 0.4×10^6^ and 0.5×10^5^ viable cells/well, respectively, and incubated at 37°C in 5% CO_2_ overnight. The medium was replaced with fresh complete hepatocyte medium supplemented with 1 µmol/l hydrocortisone hemisuccinate (SERB) and cells were maintained in this medium until incubation with FFA or cocktails.

### PCLS preparation

Steatotic liver tissue was preserved in cold preservation solution until use. Cores with a diameter of 5 mm were punched out of the tissue and placed in 5% agar (Sigma-Aldrich) at 37°C. PCLS of 250 μm thickness were cut using a Leica VT1200 S vibrating microtome in ice-cold Krebs–Henseleit buffer supplemented with 25 mmol/l d-glucose, 25 mmol/l NaHCO_3_ and 10 mmol/l HEPES (Sigma-Aldrich). PCLS were then incubated individually in William's E Medium (Life Technologies) containing 10% fetal calf serum (Eurobio), penicillin (200 U/ml)-streptomycin (200 µg/ml), fungizone (2.5 µg/ml) (Life Technologies) and insulin (0.1 U/ml) and supplemented with 25 mmol/l glucose at 37°C under continuous supply of 90% O_2_/5% CO_2_ in six-well plates with continuous shaking (90 times/min). After an hour of pre-incubation in 4 ml of the medium, the slices were transferred to new transwell inserts in 12-well plates with 2 ml of fresh medium and subsequently incubated with cocktails.

### Steatosis induction in normal PHHs

To induce steatosis in normal PHHs, cells of primary culture were incubated with various concentrations of an FFA mixture composed of oleic acid (OA) and palmitic acid (PA) at a molar ratio of 2:1, respectively, and with 1% bovine serum albumin (BSA) for 48 h at 37°C in 5% CO_2_.

### Defatting of steatotic hepatocytes and PCLS

The first cocktail (referred to as D-FAT) contained only clinically approved drugs, i.e. forskolin (20 µmol/l), L-carnitine (1 mmol/l), PPARα agonist (GW7647, 1 µmol/l) and rapamycin (200 nmol/l), all from Sigma-Aldrich. In a second cocktail, NSA (10 µM) (R&D Systems Bio-Techne), a compound in preclinical development, was added to D-FAT. Steatotic PHHs and PCLS in primary culture were incubated in serum-free medium, with or without the cocktails, for 24 h.

### Cell viability evaluation

Cell viability was determined by 3-(4,5-dimethylthiazol-2-yl)-5-(3-carboxymethoxyphenyl)-2-(4-sulfophenyl)-2H-tetrazolium (MTT) colorimetric assay, which tests the ability of viable cells to convert a soluble tetrazolium salt, MTT, into a blue formazan end product by mitochondrial dehydrogenase enzymes. After 22 h of incubation, MTT solution (0.5 mg/ml; Sigma-Aldrich) was added to each well. Then, plates were kept at 37˚C for 2 h. The medium was then discarded and DMSO was added to each well to solubilize the coloured formazan product. Absorbance was read at 550 nm on a scanning microtiter spectrophotometer plate reader (Tecan Infinite M200).

### PCLS viability evaluation

PCLS viability was determined by measuring ATP levels (de Graaf et al., 2010). Slices were collected separately and placed into 1 ml of ethanol solution [70% (*v*/*v*) containing 2 mmol/l ethylenediaminetetraacetic acid (pH 10.9)], immediately frozen and stored at −80°C until further analyses. After thawing, the slices were homogenized using the TissueLyser Instrument (Qiagen) and centrifuged (8050 ***g***). ATP content was measured in the supernatant using the ATP Assay Kit (Promega) in a white 96-well plate according to the manufacturer's protocol using the Tecan Infinite M200 plate reader and a standard ATP calibration curve. The concentration of ATP was normalized for the total protein content in the remaining sample pellet. The sample pellet was dissolved in NaOH. The protein content was estimated using the bicinchoninic acid protein assay (BCA Assay kit, Thermo Fisher Scientific) using BSA for the calibration curve.

### Oil Red O staining

Intracellular lipids were stained by Oil Red O (Sigma-Aldrich). Cells and PCLS were washed with phosphate buffered saline (PBS) and fixed with 4% paraformaldehyde in PBS for 10 min. Fixed cells were incubated with Oil Red O solution for 30 min at room temperature, then with 4′,6-diamidino-2-phenylindole (DAPI, Life Technologies) and washed with PBS. For PHHs, the fluorescence images were viewed using the Olympus IX83 microscope, acquired with CellSens V1.6 and analyzed using Fiji software. The area occupied by lipid droplets in the image is displayed by Fiji software as surface area in µm^2^, and was normalized to cell number by semi-automated counting of DAPI. For PCLS, videos images were acquired using a Leica SP2 confocal laser scanning microscope and the 3D image analysis was performed with Bitplane scientific software.

### Quantification of intracellular TG content

Intracellular lipids were extracted from PHHs using hexane/isopropyl alcohol (3:2) (Tanaka et al., 1993). Cells were washed and incubated with hexane-isopropyl alcohol (3:2, *v*/*v*) 500 µl per well in 12-well culture plates in a shaker (80 rpm/minute) at room temperature for 60 min. The contents of all wells was then transferred into a glass tube for nitrogen evaporation of the organic solvent. After evaporation, lipids were resuspended in isopropyl alcohol and transferred into duplicate 96-well plates for analysis after drying. TG were measured using enzymatic kits (Thermo Fisher Scientific). The absorbance of each well was measured using a microplate reader and converted to concentration based on a standard curve. Results were normalized to the cell protein content.

For liver tissue, TG concentrations were measured as described previously (Folch et al., 1957). Briefly, liver tissues (20-30 mg) were homogenized in 1 ml of PBS using a Tissue-Lyser Homogenizer (Qiagen) for three cycles, 30 s each. Homogenates were transferred to clear glass tubes (Labelians Group CML-ID) and then mixed with 5 ml of chloroform and methanol (2:1, vol/vol). The mixture was vortexed vigorously and incubated for 15 min on ice to allow separation into two phases. The lipid extracts were condensed at the bottom phase by centrifugation at 1650 ***g*** for 10 min at 4°C. An aliquot of the organic solvent phase was evaporated under nitrogen gas. Lipid extracts of liver tissues were dissolved in 200 μl of isopropanol with 1% Triton X-100. For the assay itself, 10 μl of TG standard or liver lipid extract was added to a 96-well plate, and 200 μl of Infinity triglyceride reagents was added to the microplate using the InfinityTM Triglyceride kit (Thermo Fisher Scientific) according to the manufacturer's instructions. The protein concentrations in the lysates were determined via the BCA Assay kit. The absorbance was measured using the Tecan Microplate Reader. Hepatic TG levels were normalized to protein content. It should be emphasized that TG content showed a high correlation with the histological scoring system for steatosis in all liver samples (Fig. S6).

### ApoB100 and ketone body secretions

Cell and PCLS supernatants were collected and centrifuged at 150 ***g*** for 5 min to remove debris. ApoB100 and ketone bodies were measured using an ELISA kit according to the manufacturer's instructions (Sigma-Aldrich). Results were normalized to the cell protein content.

### RT-qPCR

RNA extraction, reverse transcription and quantitative PCR were performed as previously described (Aoudjehane et al., 2016). Total RNA was extracted from hepatocytes and PCLS using the RNeasy minikit (Qiagen) and the TRIzol reagent, respectively, according to the manufacturer's instructions. Then, 500 ng of total RNA was reverse transcribed into cDNA using a reverse transcriptase (Promega) and real-time PCR was performed using the LightCycler 480 SYBR Green I Master Kit on an LC480 device (both from Roche Diagnostics). The mRNA level was calculated by normalizing the threshold cycle (CT) of target genes to the CT of the 28S ribosomal RNA housekeeping gene, as previously described (Livak and Schmittgen, 2001). The primers (Table S1) were designed using primer software from Roche Diagnostics and were purchased from Eurogentec.

### Western blot analysis

Cells and tissues were lysed on ice directly in Laemmli buffer (Bio-Rad) or in RIPA buffer supplemented with β-mercaptoethanol (Sigma-Aldrich). Then, 30 µg of proteins were boiled and subjected to SDS-PAGE and transferred to nitrocellulose membranes (Bio-Rad). The blots were blocked with Tris buffered saline (TBS), 0.1% Tween-20 containing 5% BSA or non-fat dry milk, and incubated overnight at 4°C with the following antibodies: anti-LC3B (CN#2775, Cell Signaling Technology, 1/1000), anti-SIRT1 (CN#8469, Cell Signaling Technology, 1/1000), anti-tubulin (CN#66031, Proteintech, 1/5000). They were then incubated with secondary HRP-link antibody (#NXA931 for mouse IgG HRP antibody or #NA934 for rabbit IgG HRP antibody, GE Healthcare Europe, 1/2000) for 1 h at room temperature. Signals were revealed by chemiluminescence (Thermo Fisher Scientific).

### ROS assay

Intracellular ROS were measured using the peroxide-sensitive fluorescent probe 2′,7′-dichlorofluorescein diacetate (H2DCFDA; Sigma-Aldrich). The cells were exposed to serum-free medium containing 50 µmol/l H2DCFDA in the dark for 60 min and then washed thrice with PBS. The fluorescence intensity was measured at 510 nm (excitation wavelength) and 605 nm (emission wavelength) using a luminometer (Tecan). Results were normalized to the cell protein content, determined by BCA Assay kit (Thermo Fisher Scientific).

### Histology of PCLS

PCLS were fixed with 10% neutral-buffered formalin (Sigma-Aldrich) at room temperature for 24 h. Fixed PCLS were embedded in paraffin and were cut into 5 μm-thick sections for Haematoxylin and Eosin (H&E) and Sirius Red staining. Stained liver tissue sections were scanned using a Pannoramic Digital Slide Scanner (Pannoramic MIDI II, 3DHISTECH) and analyzed with the manufacturer's viewer software.

### Statistical analysis

Results are expressed as mean±s.e.m. and differences between groups were tested by one-way ANOVA and the Mann–Whitney test implemented with the Prism software (Version 6.0, GraphPad Software). A *P*-value<0.05 was considered significant.

## Supplementary Material

Supplementary information
