# Supplementary material for: Novel defatting strategies reduce lipid accumulation in primary human culture models of liver steatosis
Source: Dis Model Mech. 2020 Apr 29;13(4):dmm042663. doi: 10.1242/dmm.042663 (PMC7197711; doi:10.1242/dmm.042663)

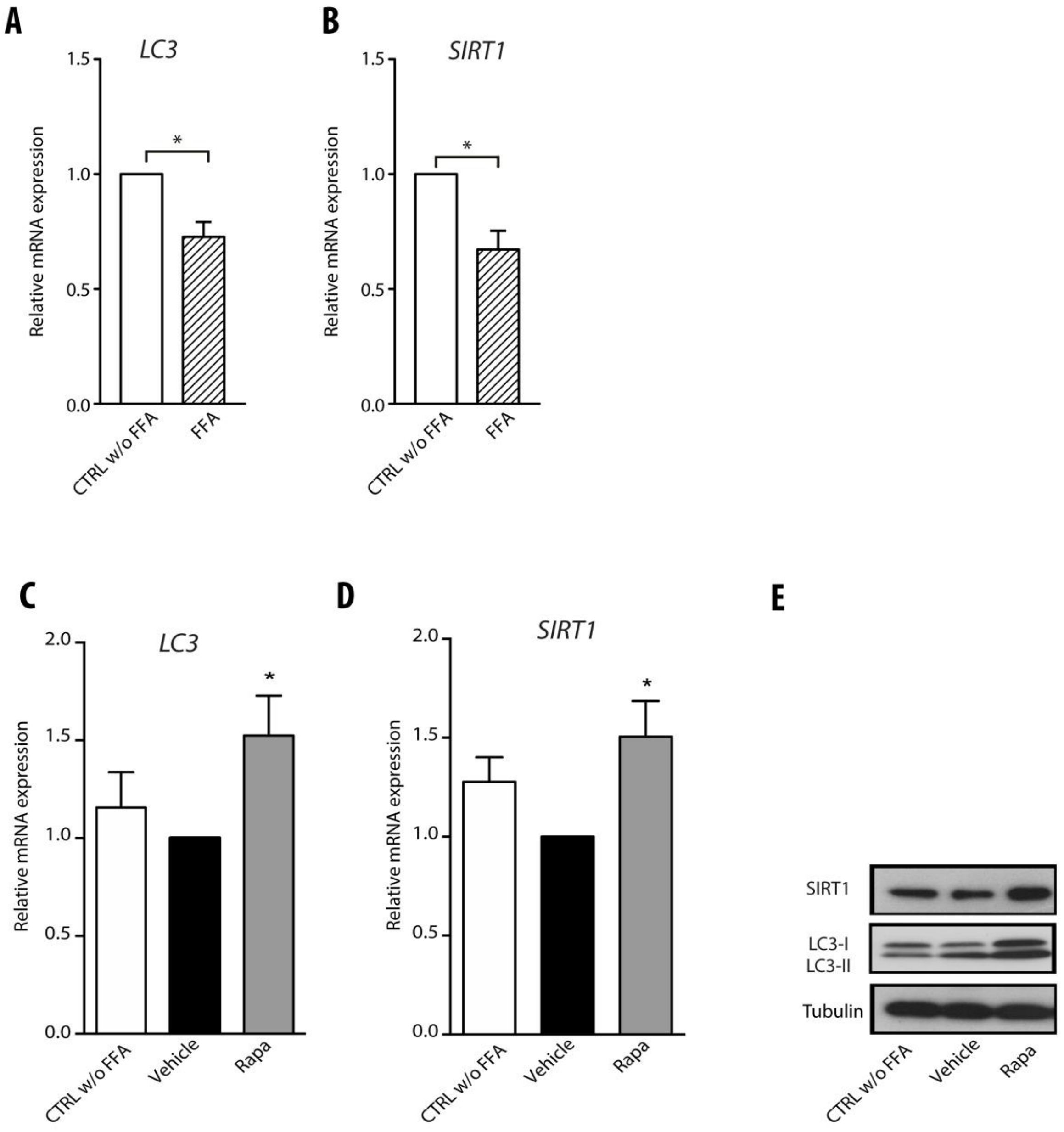

Figure S1. Steatosis induction is associated with decreased autophagy in primary human hepatocytes (PHH).

Normal human hepatocytes after 24 hours of primary culture, were incubated with or without FFA mixture (OA:PA, 500:250 µmol/L) for 48 hours and the induction of autophagy was assessed by RT-QPCR analysis **(A, B, C and D)** and immunoblots **(E)** of microtubule-associated proteins 1A/1B light chain 3B (LC3) and sirtuin-1 (SIRT1). In panels **(A, B,C and D),** means ± SEM of 6 cell preparations are shown relative to controls (CTRL); *P<0.05 *versus* CTRL (One-Way ANOVA).

Disease Models & Mechanisms • Supplementary information

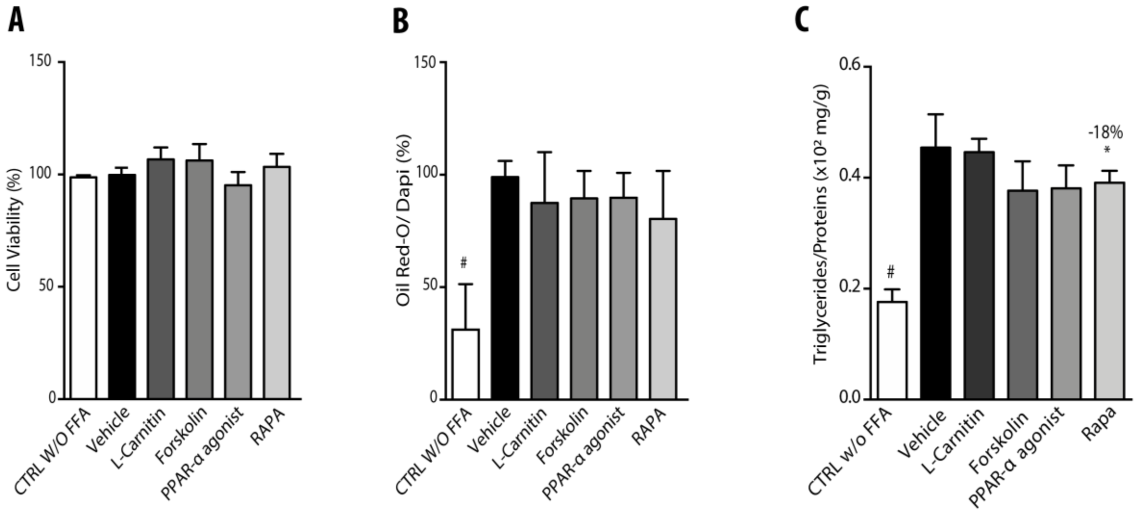

Figure S2. Components of cocktail effect on fat loaded PHH on viability and defatting. Normal human hepatocytes after 48 hours of primary culture, were incubated with FFA mixture (OA:PA, 500:250 µmol/L) for 48 hours, and then with different molecules which composed the DFAT cocktail or with the vehicle for 24 hours, and their supernatant collected for the analysis of **(A)** cell viability, assessed via the MTT assay; **(B)** lipid droplet content, assessed by Oil Red-O staining and **(C)** intracellular triglyceride (TG) content normalized for cell protein. Means ± SEM of 4 cell preparations are shown relative to vehicle. In all panels, #*P*<0.05 *versus* control, **P*<0.05 *versus* vehicle (One-Way ANOVA).

Disease Models & Mechanisms • Supplementary information

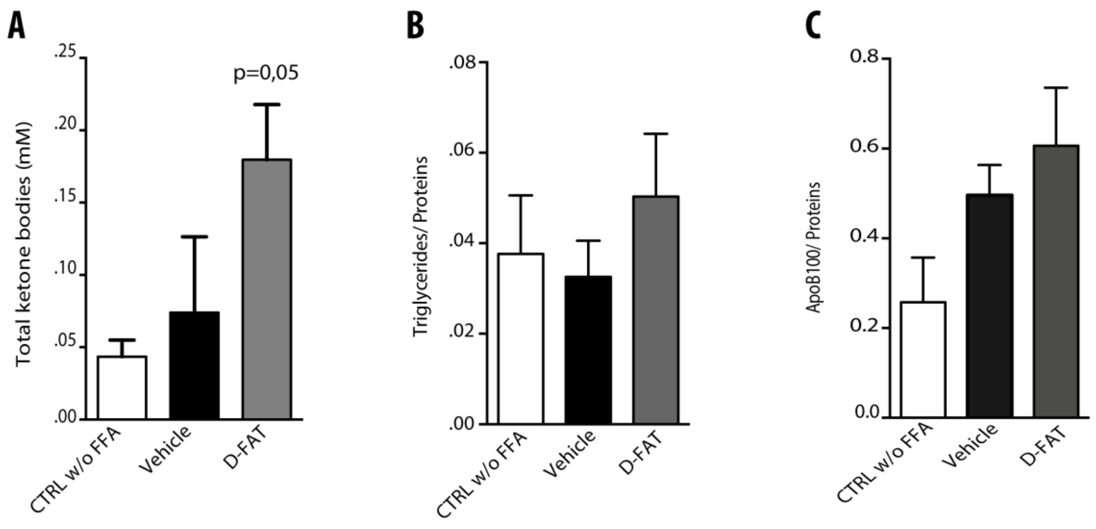

Figure S3. Changes in the supernatant of fat-loaded PHH in response to the D-FAT cocktail.

Normal human hepatocytes after 48 hours of primary culture, were incubated with FFA mixture (OA:PA, 500:250 µmol/L) for 48 hours, and then with D-FAT or vehicle for 24 hours, and their supernatant collected for the analysis of **(A)** ketone bodies in the cell supernatants **(B)** triglycerides; and **(C)** apolipoprotein B100 (ApoB100). All results were normalized for cell protein content and shown as means ± SEM of 6 cell preparations (One-Way ANOVA).

Disease Models & Mechanisms • Supplementary information

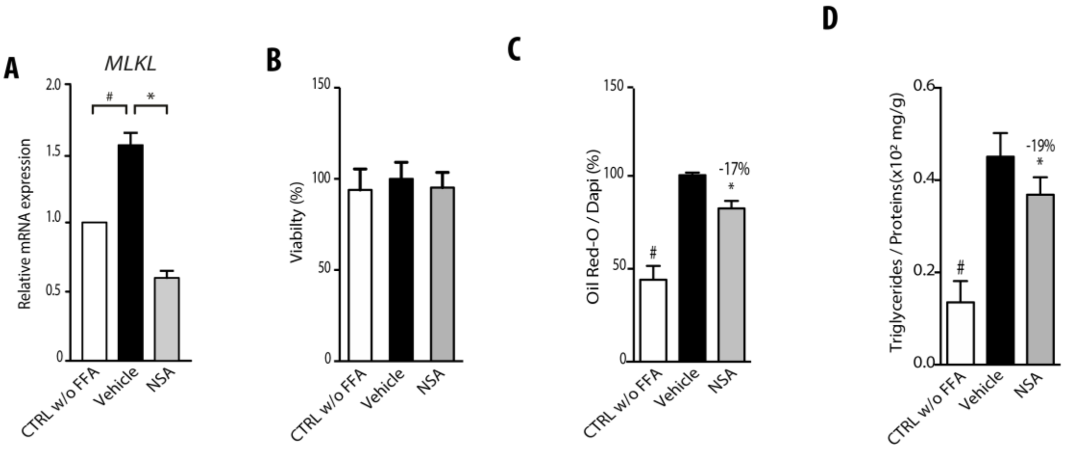

Figure S4. **Effect of necrosulfonamide (NSA) on defatting in fat-loaded PHH.**

Normal human hepatocytes in primary culture, were incubated with or without FFA mixture (OA:PA, 500:250 µmol/L) for 48 hours, and thereafter FFA-loaded PHH were treated with NSA or vehicle for 24 hours and examined for mixed-lineage kinase domain-like (MLKL) expression by RT-qPCR **(A)** cell viability, assessed by MTT assay **(B)**; lipid droplet content, assessed by Oil Red-O staining **(C)** and intracellular triglyceride (TG) content normalized for cell protein **(D)**. Means ± SEM of 6 cell preparations are shown relative to controls in B and to the vehicle in **A, C-D**. In all panels, $^{\#}P<0.05$ *versus* control, $^{*}P<0.05$ *versus* vehicle (One-Way ANOVA).

Disease Models & Mechanisms • Supplementary information

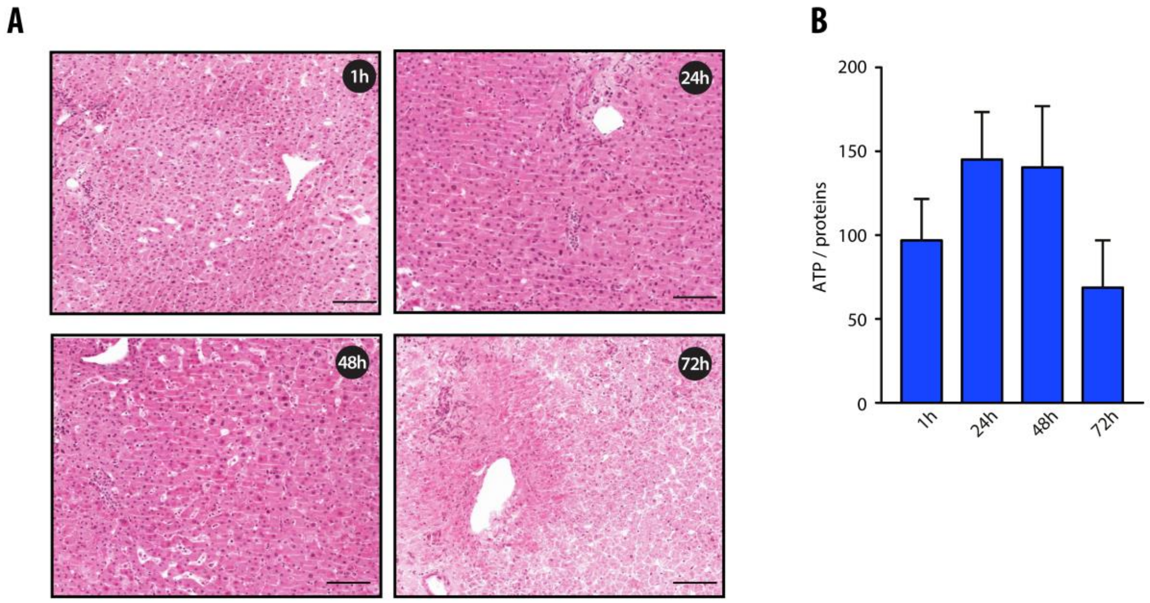

Figure S5. Time course of PCLS in culture.

PCLS were prepared from human liver samples, and after 1 hour, 24 hours, 48 hours or 72 hours in primary culture, they were examined for (A) Histology of hematoxylin-eosin-stained tissue sections; representative images are shown at magnification 20X; (B) Viability assessed by ATP content. Means ± SEM of 4 cell preparations is shown (One-Way ANOVA), Scale bar 200 µm.

Disease Models & Mechanisms • Supplementary information

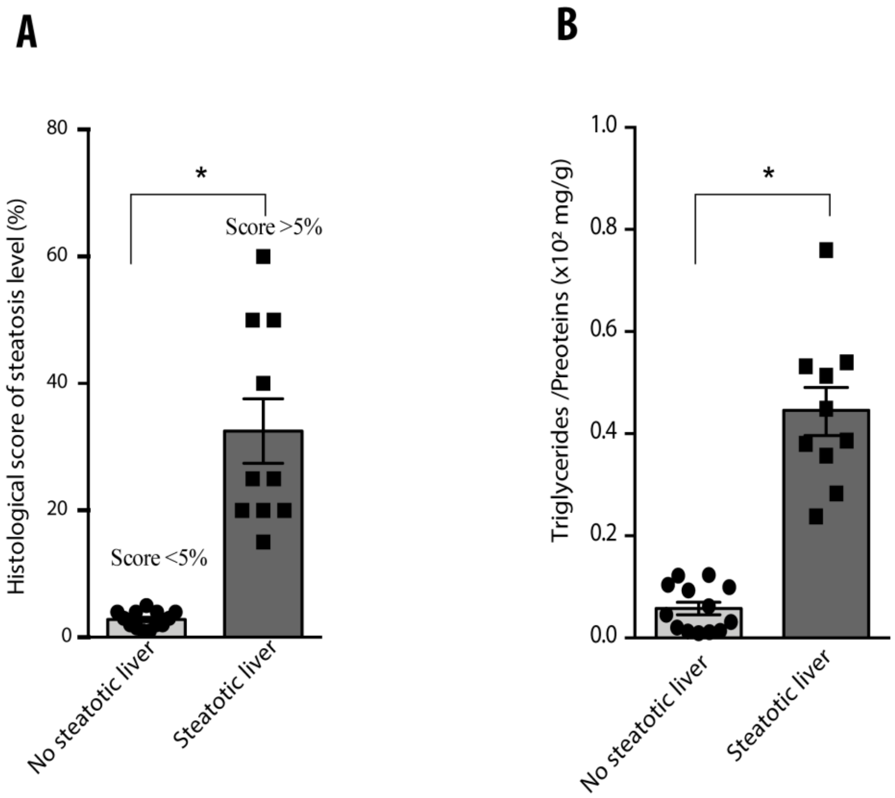

Figure S6. Histological score of steatosis and triglyceride quantification of liver samples. Histological score of steatosis (A). Triglyceride contents (B). Means ± SEM of 23 preparations (steatotic and no steatotic samples) and in all panels **P*<0.05 *versus* no steatotic samples (two-tailed Student's t-test)

Disease Models & Mechanisms • Supplementary information

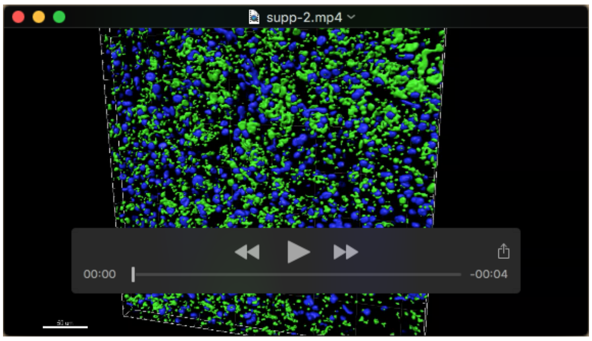

Movie 1. The live imaging videos of lipid droplet content in steatotic PCLS without treatment (vehicle) for 24 hours, and examined for lipid droplet content, assessed by Oil Red-O staining and quantified using video image analysis system.

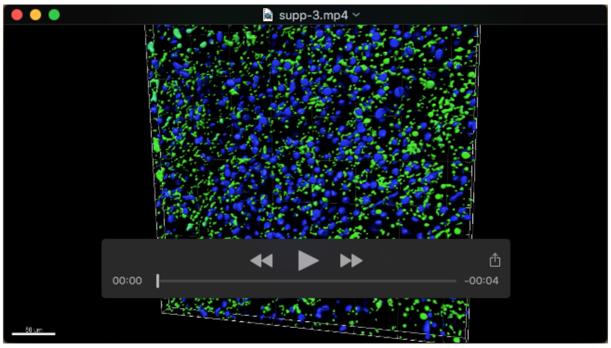

Movie 2. The live imaging videos of lipid droplet content in steatotic PCLS treated with D-FAT for 24 hours, and examined for lipid droplet content, assessed by Oil Red-O staining and quantified using video image analysis system.

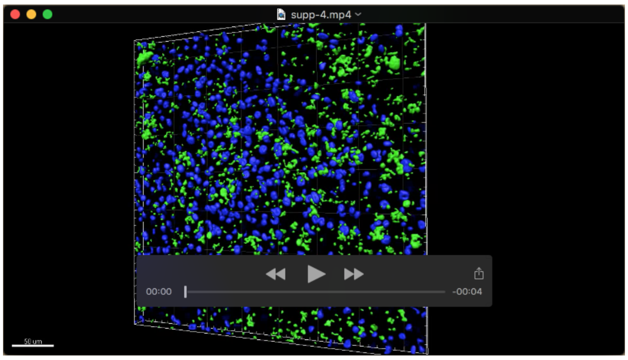

Movie 3. The live imaging videos of lipid droplet content in steatotic PCLS treated with D-FAT in combination with NSA for 24 hours, and examined for lipid droplet content, assessed by Oil Red-O staining and quantified using video image analysis system.

Table S1: Primers used for real-time PCR

Genes	Accession number	Forward primer 5'-3'	Reverse primer 5'-3'	Size product
28S	NR_003287.2	TTGAAAATCCGGGGGAGAG	ACATTGTTCCAACATGCC AG	100
ACOX1	NM_007292.5	TGCTCAGAAAGAGAAATGGC	TGGGTTTCAGGGTCATACG	132
ApoB100	NM_000384.2	GGAGCTGCTGGACATTGCTA	ATGGCAGCTTTCTGGATCAT	201
ApoA1	NM_001318017.1	CCTTGGGAAAACAGCTAAACC	CCAGAACTCCTGGGTCACA	107
CHOP	NM_001195057.1	TTGCCTTTCTCCTTCGGGAC	CAGTCAGCCAAGCCAGAGAA	178
CPT1A	NM 001031847.2	CCTCCGTAGCTGACTCGGTA	CGGAGTGACCGTGAACTGA	78
FAS	NM_004104.4	AGCTGCCAGAGTCGGAGAAC	TGTAGCCCACGAGTGTCTCG	357
GADD34	NM_014330.3	TCCTCTGGCAATCCCCCATA	TGGTTTTCAGCCCCAGTGTT	104
LC3	NM_022818.5	GAACGATACAAGGGTGAGAAG C	AGAAGGCCTGATTAGCATTGAG	133
IL-1β	NM_000576.2	CTGAGCTCGCCAGTGAAATG	CATGGCCACAACAACTGACG	200
IL8	NM_000584.3	GTGATTGAGAGTGGACCACA	CTCTGCACCCAGTTTTCCTT	101
MTTP-1	NM_001300785.1	TCAAAGTACACGGCCATTCC	GCCAGAGCTCCGAGAGAGA	82
PGC1a	NM_001330751	GTCACCACCCAAATCCTTAT	ATCTACTGCCTGGAGACCTT	131
SIRT1	NM_012238.5	TAGACACGCTGGAACAGGTTGC	CTCCTCGTACAGCTTCACAGTC	117
SREBP1	NM_004176.4	CGCTCCTCCATCAATGACA	TGCGCAAGACAGCAGATTTA	88
TNF	NM_000594.3	TCTTTCTCGAACCCCGAGTGA	CCTCTGATGGCACCACCAG	152

Disease Models & Mechanisms • Supplementary information

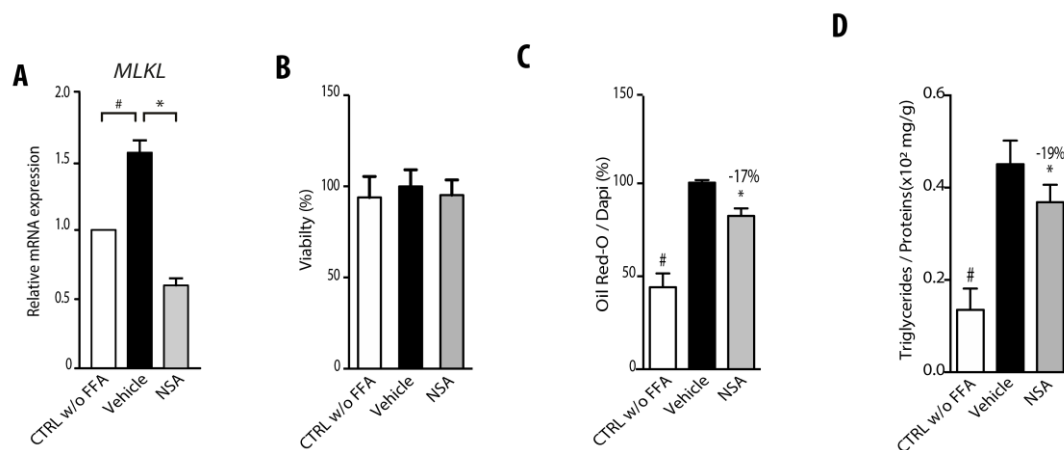

Supplement: Supplementary information [file dmm-13-042663-s1.pdf]
